# Book Review

**Published:** 1948

**Authors:** 


### Reviews of Books

**Introduction to Gastro-Enterology.** By J. D. Lickley, M.D. Pp. viii., 143. Illustrated. Bristol: John Wright & Sons Ltd. 1947. Price 8s. 6d.—The object of the author has been to produce an elementary manual on the anatomical, physiological, and clinical aspects of the alimentary tube. This has necessitated a careful selection of the more important details from the large mass of information on the subject. Such selection must, of course, introduce an individual outlook, which the author freely acknowledges. At the same time, he must be congratulated on having covered the field in fair detail. It should be of great use to those graduates who wish to get an outline of the physiology of the tract without wading through the endless papers on the subject. The diagrams are well chosen and adequate. It is a pity that no space has been found for the significance of the fractional test meal and the effects of vagus section. This is an interesting book which should supply a need.

**Dermatoses among Gas and Tar Workers.** By W. D. Jenkins, M.R.C.S., L.R.C.P. Pp. 54. Illustrated. Bristol: John Wright & Sons Ltd. 1948. Price 25s.—The author was for many years chief medical officer to a large gas company employing 6,659 personnel. Outlines of the methods used for gas and coalite manufacture, tar distillation and treatment of by-products in the chemical works are given. The monograph deals with occupational dermatoses occurring during the period 1933–42. Incapacitating dermatitis was 0.03 per cent. per annum, non-incapacitating dermatitis which did not involve loss of working time was double this figure, but still remarkably low. During the ten years under review 715 other cases of skin disorder were examined; epidermophytosis of the feet was common amongst stokers who stand on heated stage plates. In those working with tar 2.7 per cent. per annum developed papillomata on the exposed surfaces and in all the employees twenty-three cases of epithelioma occurred in the ten years. X-ray therapy was used for both types of tumour. Protective measures are discussed: the usual barrier creams proved unsatisfactory; but a Kaolin paste applied to the face was of great help in pitch workers. This monograph which contains some excellent photographs should be read by those interested in the fields of Industrial Medicine and Dermatology.

**Physical Signs in Clinical Surgery.** Part I. Eleventh Edition. By Hamilton Bailey, F.R.C.S., F.A.C.S., F.I.C.S., F.R.S.E. Pp. xi., 100. Illustrated. Bristol: John Wright & Sons Ltd. 1948. Price 8s. 6d.—Medical publishers nowadays are staggering under great difficulties, and as the preface to this book explains, the colour blockmakers are the most difficult of all. To avoid endless delays, this the eleventh edition being published in four parts, and is paper-bound. The present issue is deals with general surgery, and with injuries and diseases of the head. We can only express our sympathy. There is, however, no falling off in quality in what we are given. There are many new pictures, and the text has been revised and added to. Really rare diseases have wisely been omitted, but everything a doctor is likely to see in his years of practice, or the student in the hospital or in his examination, is included and commented on. There are 178 illustrations, many of them coloured, and all of them excellently turned out. It is not surprising that so many editions have been sold out, and that the book has been translated into seven European languages (why Bulgarian ?).

**Psychiatry.** By WILLIAM A. O'CONNOR, L.M.S.S.A. D.P.M. Pp. xi., 380. Bristol: John Wright & Sons Ltd. 1948. Price 35s.—This forceful book has been written with a purpose. It has a mission, but it must not be thought to have been written impulsively or hurriedly. The author considered his project for many years. The result is excellent. Dr. O'Connor's object is to stress the obvious, the psychological side of illness, although he approves of established beneficial physical treatments whenever required. Throughout he emphasizes the necessity of the psychological approach, even accompanying malarial and other physical therapies. Written for the beginner, this book cannot be completely understood by him. Although a helpful glossary of useful definitions is appended, some terms unlikely to be known by the novice, such as presbyophrenia, appear in the text only. This up-to-date book is more suitable for the student for a special diploma or for the psychiatrist of a few years' experience. For these, the author's views, knowledge and wisdom will be of inestimable value. They will be able to recognize and ignore the few errors that creep into the most careful of first editions. Such are met with where repression is described as pathological on one page and as not necessarily so later. Omissions are few, but amongst these are addictions to paraldehyde and to methylated spirits, the chemistry of bromide poisoning and the enuresis of the low-grade mental defective without spina bifida. That the depressive is usually more depressed in the morning is not a generally recognized observation, and the view that parents carry contradictory genes is a poetic licence and not an eugenic fact. The author's style of writing is noteworthy. The phraseology, the choice of word and the verbal visualization are masterful. The description of the deteriorating simple schizophrenic as " taking life as it comes, wholly at peace with his progressive disintegration " gives a vivid picture of the dementing schizoid. So throughout. Dr. O'Connor picks his words and forms his sentences to give the maximum effect. This book should be read by all the younger psychiatrists and especially by those trained on a physical rather than on a psychological plane. It is recommended. Reproduction is good, with clear, uncrowded print. References are not given, although some authors are mentioned by name. The index is full and detailed.

**Surgery.** By C. A. PANNETT, B.Sc., M.D., F.R.C.S. Second Edition. Pp. xiv., 769. Illustrated. London: Hodder & Stoughton Ltd. 1947. Price 27s. 6d.—Professor Pannett's book, the second edition of which now appears, is essentially one for the undergraduate. Although the medical student of to-day has a large and varied choice of surgical text-books it cannot be denied that many of these do little to ease his burden. This cannot be said of Professor Pannett's book. The basic principles of surgery are presented in a clear and concise manner with a commendable absence of side-stepping. All that the student should know is there, and written in a language which he can understand, and understand quickly. Naturally, not all will agree with some of the author's opinions ; for example, when he states that " the breast is one of the regions of the body where the surgery of malignant disease meets with considerable success." It would be more correct to say that this is one of the regions where surgery *should meet* with considerable success. But does it ? Again, in the section on the treatment of inguinal hernia one would have liked to see some mention of what is now regarded as an essential step in every hernia operation, namely the repair of the transversalis fascia. These, however, are minor criticisms which do not detract from a good book, which can be thoroughly recommended.

**Milk Products.** By W. CLUNIE HARVEY, M.D., D.P.H., and HARRY HILL, F.R.San.I. Second Edition. Pp. viii., 343. Illustrated. London : H. K. Lewis & Co. Ltd. 1947. Price 30s. net.—This is the second edition of a well-known handbook which was first published in 1937. It serves as a companion volume to the author's previous publications : " *Milk: Production and Control* " and " *Pasteurization* ". It is particularly welcome at this time when, as the authors point out in their preface, the whole nation has suddenly become " milk-conscious " for the first time in its history. The milk products dealt with include ice-cream, cream, butter (and margarine), cheese, condensed milk, evaporated milk and dried milk : and for ease of comparison each of the chapters has been compiled to a standard plan. Although dealing with a highly technical subject, the book has the merit of being easily read. The text has been brought up to date without increasing its length. The illustrations are well produced and include modern plant and machinery. The many changes in food legislation consequent upon the recent war and the establishment of a Ministry of Food have involved the authors in considerable work in bringing up to date the legislative control of the various milk products dealt with. The publishers are to be congratulated on a production which in spite of the war economy standard is excellent.

